# Serum Phospholipid Fatty Acids Levels, Anthropometric Variables and Adiposity in Spanish Premenopausal Women

**DOI:** 10.3390/nu12061895

**Published:** 2020-06-25

**Authors:** María del Pilar del Pozo, Virginia Lope, Inmaculada Criado-Navarro, Roberto Pastor-Barriuso, Nerea Fernández de Larrea, Emma Ruiz, Adela Castelló, Pilar Lucas, Ángeles Sierra, Isabelle Romieu, Véronique Chajès, Feliciano Priego-Capote, Beatriz Pérez-Gómez, Marina Pollán

**Affiliations:** 1 Department of Preventive Medicine, Public Health and Microbiology, Universidad Autónoma de Madrid (UAM), 28049 Madrid, Spain; pilar_dp@hotmail.com; 2 Department of Epidemiology of Chronic Diseases, National Center for Epidemiology, Carlos III Institute of Health, 28029 Madrid, Spain; rpastor@isciii.es (R.P.-B.); nfernandez@isciii.es (N.F.d.L.); e.ruiz@externos.isciii.es (E.R.); adela.castello@uah.es (A.C.); pmlucas@isciii.es (P.L.); angeles.sierra@hotmail.es (Á.S.); bperez@isciii.es (B.P.-G.); mpollan@isciii.es (M.P.); 3 Consortium for Biomedical Research in Epidemiology & Public Health, CIBERESP, 28029 Madrid, Spain; 4 Department of Analytical Chemistry, Annex Marie Curie Building, Campus of Rabanales, University of Córdoba, 14014 Córdoba, Spain; inma.c.n@hotmail.es (I.C.-N.); q72prcaf@uco.es (F.P.-C.); 5 Maimónides Institute of Biomedical Research (IMIBIC), Reina Sofia University Hospital, 14004 Córdoba, Spain; 6 Faculty of Medicine, University of Alcalá, Alcalá de Henares, 28871 Madrid, Spain; 7 Center for Research on Population Health, National Institute of Public Health, Cuernavaca 62100, Mexico; iromieu@gmail.com; 8 Huber Department of Global Health, Emory University, Atlanta, GA 30329, USA; 9 Section of Nutrition and Metabolism, International Agency for Research on Cancer, 69008 Lyon, France; chajesv@iarc.fr

**Keywords:** fatty acids, desaturation index, obesity, body mass index, fat

## Abstract

This study investigates the still uncertain association between serum phospholipid fatty acids (PL-FA), and anthropometric and adiposity variables. A cross-sectional study was conducted with 1443 Spanish premenopausal women. Participants answered an epidemiological and a food frequency questionnaire. Anthropometric variables were measured using a bioimpedance scale. Serum PL-FAs levels were determined by gas chromatography–mass spectrometry. The association between body mass index (BMI), weight gain, body fat percentage, visceral fat index, and waist circumference with serum PL-FAs and desaturation indices was evaluated using multivariable linear regression models. BMI was positively associated with the relative concentration of saturated fatty acids (SFAs) (β = 0.94, q-val = 0.001), and with palmitoleic, dihomo-γ-linolenic (DGLA), arachidonic (AA) and α-linolenic acids, and was inversely associated with oleic, gondoic, trans-vaccenic, linoleic and γ-linolenic acids. Total fat percentage was positively associated with DGLA and AA, and inversely with linoleic and γ-linolenic acids. Low relative concentrations of some SFAs and high levels of n-6 PUFAs were associated with greater waist circumference. While the oleic/stearic and AA/DGLA acid ratios were inversely associated with BMI, DGLA/linoleic acid ratio was positively related to almost all variables. In addition to BMI, total fat percentage and waist circumference were also associated with certain individual fatty acids.

## 1. Introduction

Obesity is a worldwide epidemic associated with cardiovascular diseases, diabetes, musculoskeletal disorders and certain types of cancer, and it is considered one of the main public health problems of the 21st century. The World Health Organization (WHO) estimated that in 2016, 40% of women aged 18 years and over were overweight and 15% obese [1]. In Spain, these percentages have increased notably in recent years. In 2014, women with obesity or overweight reached 45%, according to self-reported information [2], and it has been estimated that, if the current trend continues, 55% of women will be overweight by 2030 [3]. This situation may be even worse, since self-reported data substantially underestimate the actual body mass index (BMI) [4].

Elevated plasma free fatty acid levels are an important cause of obesity associated insulin resistance and cardiovascular disease [5]. Adipokines have also been established to play an important role in lipid metabolism and in maintaining an inflammatory state in adipose tissue and obesity. The metabolic disturbances they trigger can also lead to the development of the metabolic syndrome. Leptin acts within the central nervous system to inhibit food intake and increase energy expenditure, but it also has autocrine or paracrine actions in tissues that store triacylglycerol, where it influences the rates of lipid synthesis and degradation. Adiponectin is a protein that increases fatty acid oxidation and reduces glucose synthesis in the liver [6,7].

Metabolic syndrome describes a cluster of clinical signs (central obesity, dyslipidemia, impaired glucose metabolism, and elevated blood pressure) that regardless of cause, identifies individuals at risk of atherosclerotic cardiovascular disease and diabetes mellitus 2. In Spain, the prevalence of metabolic syndrome ranged from 12% in the EPIRCE study’s women [8], to 29% in the DARIOS study’s women [9]. This prevalence was 24.6% in women of the Metabolic syndrome and Arteries REsearch (MARE) Consortium [10], with an age-associated increase in all the cohorts. Among the Spanish subjects of this consortium with metabolic syndrome, 12% had elevated glucose, elevated blood pressure and abdominal obesity [10]. In another collaborative analysis of ten European cohort studies, the age-standardized percentage of obese women with metabolic syndrome ranged from 24% to 65% [11].

On the other hand, each type of fatty acid influences obesity in a different way. The prospective cohort Nurses’ Health Study (NHS), with 41,518 women, revealed a positive association of dietary saturated fatty acids (SFAs) with weight gain [12], and an inverse relationship between the consumption of monounsaturated fatty acids (MUFAs) and polyunsaturated fatty acids (PUFAs) and body composition [13]. The European Prospective Investigation into Cancer and Nutrition (EPIC) study showed an increased risk of weight gain during 5-years of follow-up associated with high plasma levels of elaidic acid, the main industrial trans fatty acid [14]. Regarding omega-3 and omega-6 polyunsaturated fatty acids (n-3 and n-6 PUFAs, respectively), the scientific evidence has shown that the balance between these two groups could play an important role in the prevention and management of obesity [15]. Excessive circulating levels of n-6 PUFAs (a high n-6/n-3 PUFA ratio typical of Western diets) are associated with obesity and weight gain in both animal and human studies, while high levels of n-3 PUFAs (a lower n-6/n-3 PUFA ratio) contributes to decrease the risk [16].

Desaturation indices are used as markers of endogenous fatty acid metabolism. SCD-16 (ratio between palmitoleic acid and palmitic acid) and SCD-18 (oleic acid to stearic acid ratio) are biomarkers of the stearoyl-CoA desaturase 1 (SCD-1) activity [17], which converts SFAs to MUFAs. FADS1 (ratio between arachidonic acid (AA) and dihomo-γ-linolenic acid (DGLA)) is an indicator of the Δ5-desaturase activity, and FADS2 (ratio of DGLA to linoleic acid) is an indicator of the activity of Δ6-desaturase and elongase [18]. Few epidemiological studies have evaluated the association between these desaturation indices and indicators of obesity. SCD-16 has been positively associated with body mass index [19,20,21], waist circumference [19,21] and body fat [20,21]. SCD-18 has been positively related to BMI and body fat [20]. FADS1 has been inversely related to waist circumference, BMI and body fat and, finally, FADS2 has been positively associated with all of these indicators.

Most studies have evaluated the association of fatty acids with health-related outcomes using food frequency questionnaires. To overcome the limitations of this methodology, the use of biomarkers of dietary intake has been proposed. However, only essential n-3 PUFAs, n-6 PUFAs and trans-FAs are good biomarkers of dietary intake, since they cannot be endogenously synthesized. Serum phospholipid fatty acid (PL-FA) levels can be good biomarkers of dietary exposure, and the internal transformation of these fatty acids [22]. In addition, they also have the advantage of being less prone to systematic and random errors [23]. Therefore, the objective of this study is to evaluate the association between the relative concentrations of serum PL-FAs, and the desaturation indices, with anthropometric and adiposity measures in Spanish premenopausal women.

## 2. Materials and Methods

### 2.1. Study Population

In 2012, the DDM-Madrid cross-sectional study was launched with the aim of evaluating the association between vitamin D levels and mammographic density in 1466 premenopausal women (39–50 years) who underwent their routine gynecological examination at the Madrid Medical Diagnostic Center (Madrid Salud). Women were contacted by phone before their visit to the medical center to invite them to participate, and 88% agreed to collaborate. The same day that women attended their medical examination, they signed an informed consent and answered a standardized epidemiological questionnaire, administered by trained interviewers. This questionnaire collected sociodemographic information, reproductive, family and personal history, and smoking, alcohol and physical activity habits. Finally, participants completed a 117-item semi-quantitative food frequency questionnaire with information on eating habits for the previous 12 months, and that has been previously validated in several Spanish adult populations [24].

Participants’ height and weight were measured using a certified bioimpedance scale (Tanita SC-330), which estimates body fat, muscle mass, bone mass, and visceral fat. Following a standardized protocol, interviewers also measured the women’s waist and hip circumference. All these variables were measured twice, with a third measure if the first two were discrepant. Additionally, they drew a blood sample from each woman, which was subsequently centrifuged, aliquoted and stored at −80° in the biobank of the Carlos III Institute of Health. The study was conducted in accordance with the Declaration of Helsinki, and the protocol was approved by the Ethics and Animal Welfare Committee of this institute (CEI PI 02_2012_v2).

### 2.2. Analysis of Serum PL-FAs

Aliquots of 100 μL of serum were deproteinized by the addition of 300 μL of methanol 0.1% (*v*/*v*) formic acid. The mixture was vortexed for 5 min and incubated at −20 °C for 5 min. Then it was centrifuged at 4 °C for 10 min at 2000× *g* to isolate the supernatant. PL-FAs were purified and extracted using 30 mg HybridSPE^®^cartridges from Supelco (Bellefonte, PA, USA) in a vacuum manifold with disposable liners (Supelco, Bellefonte, PA, USA). The sorbent was washed twice with 500 μL of acetonitrile containing 1% (*v*/*v*) formic acid. PL-FAs were eluted by changing pH using 1 mL of methanol with 5% (*v*/*v*) ammonium hydroxide. Finally, the extract was derivatized with 1 mL of methanolic potassium hydroxide at room temperature to convert PL-FAs into their more volatile fatty acid methyl ester derivatives (FAMEs), essential for gas chromatography–mass spectrometry (GC-MS). The mixture was vortexed for 1 min and stood 10 min to complete derivatization. Then, 1 mL of hexane was added, and the biphasic system was vortexed for 5 min. The upper phase, which contains the PL-FAMEs, was evaporated and the residue was reconstituted with 50 μL of hexane before injection into the GC–MS system (Agilent 7890B-5977A, Santa Clara, CA, USA) furnished with a SP^TM^ 2560 fused silica column factor (100 m × 0.25 mm × 0.25 μm film thickness) from Supelco. Injection of the sample (1 µL) was performed in splitless mode and the injector temperature was 250 °C. The gas flow was 0.6 mL min^−1^ and the oven temperature gradient was programmed as follows: initial temperature 100 °C, hold for 5 min; ramp, 4 °C min^−1^ to 240 °C, hold for 20 min. The total analysis time was 60 min. The single quadrupole mass spectrometer was operated in the scan mode, with the instrumental parameters set at 250, 250, and 180 °C for transfer line, source, and quadrupole, respectively. The electron energy was adjusted to 70 eV and data acquisition was set from 45 to 750 *m*/*z*.

The NIST Mass Spectral Search Program v.11.0 (NIST, Washington, DC, USA) was used for spectral search (Mainlib and Replib libraries). Tentative identification was reported when the correlation between the experimental and database spectra was greater than 0.75 in the normal search mode. Confirmatory analysis was performed by analysis of a FAMEs multi-standard from Sigma–Aldrich.

The relative concentration of each PL-FA, expressed as percentage of total PL-FAs in serum, was quantified by integrating the area under the peak and dividing the result by the total PL-FAs area. The variability of the determination, expressed as variation coefficient in percentage, ranged from 0.3 to 14.9%. The relative concentration of the following 21 individual PL-FAs was determined: SFAs (14:0, 15:0, 16:0, 17:0, 18:0 and 20:0), cis-MUFAs (16:1 n-7, 17:1, 18:1 n-9 and 20:1 n-9), ruminant trans-fatty acids (16:1 n-7 and 18:1 n-7), industrial trans-fatty acids (18:1 n-9), n-3 PUFAs (18:3, 20:5 and 22:6) and n-6 PUFAs (18:2, 18:3, 20:2, 20:3 and 20:4). The ratio of palmitoleic acid (C16:1n7) to palmitic acid (C16:0) (SCD-16) and the ratio of oleic acid (C18:1n9) to stearic acid (C18:0) (SCD-18) were also analyzed. Finally, the FADS1 index -ratio between AA (C20:4 n-6) and DGLA (C20:3 n-6)- and the FADS2 index-DGLA to linoleic acid (C18:2 n-6) ratio- were determined.

### 2.3. Statistical Methods

After excluding those women whose relative serum PL-FA concentrations could not be determined, the final sample consisted of 1443 participants (98%). Means and standard deviations were calculated for continuous variables, while categorical variables were summarized with number of women and percentages.

To analyze the association between relative concentrations of PL-FAs and anthropometric variables (BMI, weight gain since age 18, body fat percentage, visceral fat index, and waist circumference), the 20th percentile and the 80th percentile of the distribution of each PL-FA was calculated. Multivariable linear regression models were used to estimate the change in the anthropometric variable when comparing women in the 80th percentile with women in the 20th percentile of each PL-FA distribution. BMI was also analyzed by tertiles. These models were adjusted for those variables that were associated with total SFAs, total cis-MUFAs, total trans-fatty acids, total n-3 PUFAs or total n-6 PUFAs: age, education (primary school or less; secondary school; university degree), smoking (non-smoker, former smoker, current smoker), alcohol consumption (non-drinker; <10 g/day; ≥10 g/day), physical activity quantified in metabolic equivalent hours per week (total MET-hours/week), hypercholesterolemia (no; yes but not treated; treated with statins) and energy intake (kcal/day). In analyses of the association with weight gain, body fat percentage, visceral fat index, and waist circumference, the models were further adjusted by BMI, in order to assess the effect, not attributable to obesity, that PL-FAs exert on these variables. To account for multiple testing, *p*-values were adjusted using the false discovery rate proposed by Benjamini and Hochberg [25]. Statistical analyses were performed using the statistical software package STATA/MP 14.0.

## 3. Results

Table 1 shows the general characteristics of the 1443 participating women. The average age was 44 years. Regarding anthropometric variables, mean BMI was 24 kg/m^2^. The mean waist circumference was 80 cm. The mean weight gain since age 18 was 8.4 kg. The body fat percentage of 30%, and the mean visceral fat index 5.2. Furthermore, 38% of women had never smoked, and most participants attended university (61%), had two or more children (53%), had used oral contraceptives (58%), consumed less than 10g/day of alcohol (66%), practiced physical activity with a mean of 11 MET-hours/week, and had a mean caloric intake of 1965 kcal/day.

Table 2 shows the mean percentage of the main serum PL-FA groups according to BMI, weight gain since age 18, body fat percentage, visceral fat index, and waist circumference. The mean relative concentration of SFAs, cis-MUFAs and trans-fatty acids varied significantly by category of all the anthropometric variables studied. While an increasing trend was observed with the mean concentration of SFAs in all variables, a decreasing trend was observed with the relative concentrations of cis-MUFAs and trans-fatty acids. On the other hand, the relative levels of n-6 PUFAs decreased with BMI and with the visceral fat index.

Table 3 shows the mean percentage of all serum PL-FAs as well as their association with BMI, by tertiles of fatty acids and comparing the 80th percentile with the 20th percentile of each fatty acid distribution. SFAs constituted 55% of the total PL-FAs. Within this family, palmitic acid (33%) and stearic acid (21%) were the most common. Within the cis-MUFA family (9% of total PL-FAs), oleic acid was the most abundant (9%). Trans-fatty acids represented 1.4% of the total. The n-6 PUFAs represented 31%, with linoleic acid (20%) and AA (9%) being the most common in this group. The composition of the n-3 PUFAs barely reached 4%, and the geometric mean of the n-6/n-3 PUFA ratio was 9.1. BMI was positively associated with relative concentrations of total SFAs (β = 0.94, q-val = 0.001), mainly due to the most common palmitic (β = 0.44, q-val = 0.011) and stearic acids (β = 0.85, q-val = 0.001) and, to a lesser extent, myristic acid (β = 0.54, q-val = 0.002). On the other hand, BMI was inversely associated with relative concentrations of cis-MUFAs (β = −0.85, q-val = 0.001), specifically with oleic acid (β = −0.90, q-val = 0.001) and gondoic acid (β = −0.48, q-val = 0.006), and with trans-vaccenic acid (β = −0.94, q-val = 0.001). Relative concentrations of total n-6 PUFAs were also slightly associated with BMI (β = −0.43, q-val = 0.048), mainly due to the effect of linoleic acid, while other individual n-6 PUFAs were positively associated (DGLA and AA). Among n-3 PUFAs, α-linolenic acid was associated with higher BMI (β = 0.93, q-val = 0.001).

The association of serum PL-FAs with the other anthropometric characteristics is shown in Table 4. After adjusting for BMI, neither weight gain during adulthood nor visceral fat index were related to PL-FAs. However, body fat percentage presented an inverse association with linoleic acid (β = −0.59, q-val = 0.007) and γ-linolenic acid (β = −0.42, q-val = 0.037), and was positively associated with DGLA (β = 0.68, q-val = 0.007) and AA (β = 0.48, q-val = 0.042). On the other hand, high relative concentrations of serum SFAs were associated with a smaller waist circumference (β = −0.86, q-val = 0.019), mainly due to the action of stearic (β = −1.20, q-val = 0.003) and arachidic acids (β = −1.24, q-val = 0.003). In contrast, the relative concentrations of total n-6 PUFAs were positively related (β = 1.02, q-val = 0.003) to waist circumference, thanks to the action of DGLA (β = 1.33, q-val = 0.003) and AA (β = 1.10, q-val = 0.003).

Table 5 shows the association between desaturation indices and obesity indicators. As it can be seen, the SCD−18 index (β = −0.95, q-val = 0.001) and the FADS1 index (β = −1.02, q-val = 0.001) were negatively associated with BMI. In contrast, SCD-16 (β = 0.51, q-val = 0.001) and FADS2 (β = 2.24, q-val = 0.001) were positively associated. This last desaturation index was also positively related to body fat percentage (β = 0.88, q-val = 0.002), visceral fat index (β = 0.13, q-val = 0.002) and waist circumference (β = 1.04, q-val = 0.002).

## 4. Discussion

This study analyzes the association between relative concentrations of serum PL-FAs, desaturation indices, and certain anthropometric variables in a sample of Spanish premenopausal women. Our results show positive associations between the relative serum concentrations of total SFAs and BMI, and between total n-6 PUFAs and waist circumference. In contrast, BMI was inversely related to the relative concentrations of cis-MUFAs, ruminant trans-fatty acids and total n-6 PUFAs. Waist circumference showed an inverse association with total SFAs. Regarding desaturation indices, we found that SCD-18 and FADS1 were inversely associated with BMI, while FADS2 was positively associated with all anthropometric variables.

Regarding serum PL-FA levels in our population, it is worth mentioning the high relative concentration of SFAs in our participants (representing 55% of the total, Table 3), which is much higher than that detected in the Spanish women in the EPIC study (39%) [26] or the reported in Mexican [19], French [27] or New York women (around 40%) [28]. Furthermore, our n-6/n-3 PUFA ratio (9.12) is higher than that estimated in the EPIC study (8.3) [26], possibly reflecting a greater adherence to the Western diet by our participants. Spain has also been described as one of the European countries with the lowest blood levels of eicosapentaenoic acid (EPA) and docosahexaenoic acid (DHA) [29].

The positive association detected between SFAs and BMI (Table 3) has been previously described [30]. Specifically, stearic acid has been associated with this measure in Mexican [19] and Lebanese [30] women. Similar results were also found in a meta-analysis of 35 randomized controlled trials [31], which showed that the intake of myristic and palmitic acids influenced the lipid profile, increasing the concentrations of total cholesterol, low-density lipoprotein (LDL) and high-density lipoprotein (HDL), while stearic acid had a lesser hypercholesterolemic effect. Furthermore, it has been described that SFAs can cause abdominal obesity due to impaired insulin sensitivity, although the results are inconclusive [32]. However, it should be noted that, although lack of insulin sensitivity is mainly related to visceral adiposity, a negative association between relative SFA levels and waist circumference was observed in this study.

On the other hand, the negative association between the relative concentration of cis-MUFAs and BMI showed in Table 3, mainly due to the action of oleic acid, is consistent with the negative trend found in a study conducted in Mexican women [19]. According to Paniagua et al., diets rich in MUFAs prevent the postprandial decrease in peripheral gene expression of adiponectin [33]. Adiponectin, one of the cytokines secreted by adipose tissue that regulates energetic metabolism through glucose and insulin interactions, stimulates the oxidation of fatty acids, reduces plasma triglycerides and improves glucose metabolism by increasing insulin sensibility [34].

Regarding the relative concentration of n-6 PUFAs, our results show that linoleic acid is negatively associated with BMI (Table 3) and with body fat percentage, unlike DGLA and AA, which are positively associated with these two variables and with waist circumference (Table 4). High relative serum DGLA levels have also been associated with obesity, body fat accumulation, and waist circumference in other studies [19]. This positive association has been attributed to AA metabolites, which play an important role in the terminal differentiation of preadipocytes to mature adipocytes [35]. DGLA is metabolized to AA, which promotes body fat gain through mechanisms related to adipogenesis, lipid homoeostasis, browning of adipose tissue, systemic inflammation, and the brain-gut-adipose tissue axis [16]. On the other hand, elevated serum DGLA levels are usually associated with an excessive intake of linoleic acid, since both fatty acids are also metabolically interconnected. However, they have an opposite association with BMI and with the body fat percentage in our study. This differential biological effect has already been described in recent studies [36].

Within the n-3 PUFA family, α-linolenic acid showed a positive association with BMI. However, this variable was not associated with the relative levels of total n-3 PUFAs or with the n-6 / n-3 PUFA ratio (Table 3). Although there are previous studies that have shown an inverse association [16], its anti-obesity effect, especially in humans, is still under discussion [37,38]. In fact, published studies show that n-3 PUFAs supplements alone do not cause changes in body weight. However, in combination with dietary interventions and/or physical exercise, they can contribute to attenuate weight gain [37].

An inverse relationship between SCD-18 and BMI was found in Table 5. This desaturation index accurately reflects hepatic SCD-1 activity, which converts SFAs into MUFAs to prevent intracellular accumulation of SFAs capable of initiating processes of lipoapoptosis [39]. Increased expression and activity of SCD-1 has been linked to insulin resistance and obesity. Experimental studies have shown that SCD-1 inhibition decreases lipogenesis and increases GLUT4-mediated glucose uptake in skeletal muscle [40]. However, the evidence is less convincing in humans [41]. While Vinknes et al. found a positive association of SCD-18 with BMI and body fat [20], Aglago et al. [19] detected a borderline inverse association with BMI. Wirfalt et al. [21] also found an inverse relationship between SCD-18 and these variables, but failed to achieve statistically significance. Although both SCD-16 and SCD-18 are biomarkers of the SCD-1 expression, both our study and previous ones have described opposite associations [19,20,21]. Since the palmitoleic acid content in the diet is considerably lower than the oleic acid content, some authors have concluded that SCD-16 is a more reliable marker of SCD-1 expression than SCD-18, which is susceptible to encompass a higher dietary component [19,42,43]. Furthermore, it has been described that the hepatic SCD-1 activity can be modified with high carbohydrate diets, dietary energy restriction, alcohol consumption, hyperinsulinemia, estrogens or physical exercise [17].

Δ5-desaturase (which converts DGLA to AA) and Δ6-desaturase (which converts linoleic acid to DGLA) are two enzymes encoded by the genes *FADS1* and *FADS2,* respectively [18]. Alterations in Δ5-desaturase and Δ6-desaturase activity have been associated with several disorders, such as metabolic derangements, insulin resistance and diabetes, or AA-derived pro-inflammatory effects [18]. Our results reflect an inverse association of FADS1 index with BMI and a positive association of FADS2 index with all the adiposity variables studied (Table 5). In relation to BMI, our results are in agreement with those reported in previous studies [21,44,45]. Regarding the other variables, Wirfält et al. also detected an inverse association of FADS1 with waist circumference and body fat percent, as well as a strong positive correlation between these variables and FADS2 [21]. Abdominal obesity has also been positively associated with Δ6-desaturase and inversely associated with Δ5-desaturase in another population-based cohort study [46].

It is known that peroxisome proliferator-activated receptors (PPARs) play an important role in regulating glucose and lipid metabolism, contributing to the development of various metabolic diseases. In particular, PPARα and PPARβ/δ play a central role in the oxidation of fatty acids, as well as in the improvement of lipid and cholesterol profiles, which reduces adiposity and prevents the development of obesity, while PPARγ contributes to energy storage by enhancing adipogenesis [47,48].

In view of these results, we can emphasize the individual importance of each PL-FA on obesity. In general, there is insufficient information on the association between fatty acid biomarkers, their endogenous synthesis, and obesity. The greatest strengths of our study lie in the characteristics of the women and in the large sample size. This is the first epidemiological study that explores the association of serum PL-FAs, analyzed individually, with anthropometric and adiposity variables in Spanish premenopausal women.

Our study also has a number of limitations. First, it is a cross-sectional study. Therefore, causal relationships between lipid levels and anthropometric variables cannot be established, and reverse causation cannot be discarded. Second, these women were recruited in a single center in Madrid and, although they come from all over the province, this limits the ability to generalize the study results. Another limitation is that the serum fatty acid composition was evaluated only once, so levels can be influenced by changes in eating habits. Furthermore, these serum fatty acid concentrations reflect short/medium-term intakes. However, this biomarker has the advantage of not depending on self-reported dietary habits, which can be affected by systematic and random errors [49]. BMI was included as a possible confounder in the analysis of other anthropometric characteristics and, given the correlation between BMI and these other variables, a certain degree of overfitting is expected. Finally, the present study is limited by the lack of information on certain biological markers, such as triglycerides, insulin, or C-reactive protein, or other lifestyle-related variables or dietary factors that may interfere with the associations found. Regarding this last point, it is worth noting the possible effect of certain preparations of isoflavones, calcium, vitamin D and insulin, since it has been observed that these could modify the metabolic parameters and body composition of women [50].

Although, in general, our results show that BMI is positively associated with relative concentrations of total SFAs and inversely with MUFA and n-6 PUFA levels, that waist circumference is inversely related to total SFAs, or that certain desaturation indices are associated with the anthropometric variables studied, our study emphasizes the importance of analyzing serum PL-FAs individually, since these, even belonging to the same family group, can have a divergent impact on certain indicators of obesity. These results could help to better understand the still not well-known relationship between fatty acids and the determinants of obesity, a health-related problem whose prevalence has increased significantly among women in Madrid in the last 20 years (37.6%) [51].

## Figures and Tables

**Table 1:** Descriptive characteristics of the study population.

	Mean (SD) or N (%)	
Age	44.3	(2.8)
Weight (kg)	63.7	(11.7)
Height (cm)	161.8	(5.8)
Body mass index (kg/m^2^)	24.3	(4.3)
Waist circumference (cm)	80.0	(11.2)
Hip circumference (cm)	98.2	(9.3)
Waist-to-hip ratio	0.8	(0.1)
Weight at age 18 (kg)	54.6	(7.5)
Weight gain since age 18 (kg)	8.4	(10.2)
Body fat percentage	30.3	(7.3)
Visceral fat index	5.2	(2.4)
Physical activity, total MET-hours/week	11.3	(17.0)
Total energy intake (kcal/day)	1965.4	(590.5)
Education		
Primary school or less	65	(4.5)
Secondary school	494	(34.3)
University	882	(61.2)
Age at menarche	12.5	(1.4)
Age at first birth	31.1	(4.7)
Number of children		
None	341	(23.6)
1	336	(23.3)
2	683	(47.3)
>2	83	(5.8)
Cumulative lactation (months)		
No	84	(7.6)
1–6	364	(33.1)
7–12	334	(30.3)
>12	319	(29.0)
Use of oral contraceptives		
Never	549	(38.3)
Past use	836	(58.4)
Current use	47	(3.3)
Tobacco consumption		
No	552	(38.3)
Former smoker	503	(34.9)
Current smoker	388	(26.9)
Alcohol consumption (g/day)		
No	255	(20.0)
<10	839	(66.0)
≥10	178	(14.0)
Hypercholesterolemia		
No	1243	(87.0)
Yes, not treated	153	(10.7)
Treated with statins	32	(2.2)

Values are mean (standard deviation) for continuous variables and number of women (percentage) for categorical variables.

**Table 2:** Relative concentrations of serum phospholipid fatty acids according to anthropometric characteristics.

		SFAs		cis-MUFAs		trans-FAs		n-6 PUFAs		n-3 PUFAs	
	N	Mean (SD)	*p*-Val	Mean (SD)	*p*-Val	Mean (SD)	*p*-Val	Mean (SD)	*p*-Val	Mean (SD)	*p*-Val
Body mass index (kg/m^2^)											
<18.5	26	53.27 (4.13)	<0.001	10.17 (1.59)	<0.001	1.53 (0.24)	<0.001	31.72 (4.13)	0.014	3.31 (1.19)	0.729
18.5–24.9	945	54.44 (4.77)		9.58 (2.07)		1.45 (0.27)		30.95 (4.07)		3.58 (1.30)	
25–29.9	331	55.25 (5.36)		9.05 (2.72)		1.41 (0.29)		30.64 (4.77)		3.64 (1.30)	
≥30	140	56.51 (4.50)		8.62 (1.80)		1.30 (0.29)		30.15 (3.53)		3.42 (1.07)	
Waist circumference ^a^ (cm)											
<74.35	476	54.47 (4.43)	0.011	9.74 (2.08)	<0.001	1.45 (0.26)	<0.001	30.77 (3.99)	0.904	3.58 (1.36)	0.788
74.35–83.01	473	54.65 (4.78)		9.35 (1.83)		1.44 (0.27)		30.99 (3.92)		3.57 (1.25)	
>83.01	474	55.27 (5.46)		9.04 (2.67)		1.39 (0.30)		30.74 (4.68)		3.56 (1.23)	
Weight gain since age 18 ^a^ (kg)											
<4.0	412	54.19 (4.56)	0.001	9.71 (2.05)	<0.001	1.46 (0.27)	<0.001	31.10 (3.97)	0.103	3.54 (1.36)	0.547
4.0–11.2	404	54.66 (5.02)		9.46 (2.12)		1.44 (0.27)		30.89 (4.24)		3.55 (1.22)	
>11.2	408	55.31 (5.01)		9.09 (2.63)		1.39 (0.29)		30.62 (4.45)		3.60 (1.27)	
Body fat percentage ^a^											
<27.1	458	54.22 (4.66)	<0.001	9.78 (2.10)	<0.001	1.46 (0.27)	<0.001	31.02 (4.13)	0.127	3.53 (1.37)	0.798
27.1–33.4	461	54.64 (4.62)		9.37 (1.98)		1.44 (0.26)		30.95 (3.89)		3.60 (1.18)	
>33.4	454	55.55 (5.46)		8.94 (2.39)		1.38 (0.30)		30.59 (4.68)		3.55 (1.24)	
Visceral fat index ^a^											
<5	613	54.20 (4.63)	<0.001	9.64 (1.95)	<0.001	1.46 (0.27)	<0.001	31.12 (4.07)	0.021	3.59 (1.36)	0.947
5–6	453	54.87 (5.09)		9.43 (2.59)		1.43 (0.27)		30.77 (4.21)		3.49 (1.17)	
7–18	306	55.88 (5.22)		8.71 (1.84)		1.36 (0.31)		30.45 (4.61)		3.61 (1.22)	

Values are expressed as percentage of total serum phospholipid fatty acids. ^a^ In tertiles.

**Table 3:** Mean percentage of serum phospholipid fatty acids and their association with body mass index.

	Total	Tertiles of Serum PL-FAs ^a^		80th vs. 20th Percentiles ^b^	
		Tertile 2	Tertile 3		
	Mean (SD)	β (95% CI) ^c^	β (95% CI) ^c^	β (95% CI) ^c^	q-Val ^d^
SFAs					
14:0 myristic acid	0.20 (0.16)	0.35 (−0.20; 0.90)	1.48 (0.92; 2.04)	0.54 (0.24; 0.85)	0.002
15:0 pentadecanoic acid	0.09 (0.05)	0.49 (−0.07; 1.05)	0.46 (−0.11; 1.02)	−0.00 (−0.31; 0.30)	0.985
16:0 palmitic acid	32.92 (1.97)	0.65 (0.10; 1.20)	1.36 (0.80; 1.92)	0.44 (0.12; 0.75)	0.011
17:0 margaric acid	0.26 (0.46)	0.02 (−0.55; 0.58)	−0.34 (−0.90; 0.23)	−0.02 (−0.07; 0.02)	0.407
18:0 stearic acid	21.21 (4.33)	0.43 (−0.13; 0.99)	0.96 (0.40; 1.52)	0.85 (0.40; 1.29)	0.001
20:0 arachidic acid	0.12 (0.06)	0.20 (−0.35; 0.76)	0.53 (−0.03; 1.09)	0.32 (−0.11; 0.74)	0.211
Total saturates (SFAs)	54.81 (4.91)	0.27 (−0.28; 0.82)	1.18 (0.62; 1.74)	0.94 (0.52; 1.36)	0.001
cis-MUFAs					
16:1 n-7 palmitoleic acid	0.32 (0.17)	0.20 (−0.35; 0.76)	1.09 (0.53; 1.65)	0.53 (0.24; 0.83)	0.001
17:1 heptadecenoic acid	0.02 (0.12)	-0.22 (−0.77; 0.34)	0.39 (-0.17; 0.94)	−0.01 (−0.05; 0.03)	0.632
18:1 n-9 oleic acid	8.96 (2.17)	−1.09 (−1.64; −0.54)	−1.98 (−2.53; −1.42)	−0.90 (−1.19; −0.61)	0.001
20:1n-9 gondoic acid	0.07 (0.03)	−0.82 (−1.38; −0.27)	−1.09 (−1.64; −0.53)	−0.48 (−0.79; −0.16)	0.006
Total cis-MUFAs	9.37 (2.23)	−0.85 (−1.40; −0.30)	−1.84 (−2.39; −1.28)	−0.85 (−1.15; −0.56)	0.001
trans-fatty acids					
Ruminant trans-fatty acids					
16:1 n-7 palmitelaidic acid	0.13 (0.05)	−0.43 (−1.00; 0.13)	−0.44 (−1.00; 0.12)	−0.17 (−0.54; 0.19)	0.410
18:1 n-7 vaccenic acid	1.16 (0.24)	−1.20 (−1.75; −0.64)	−1.26 (−1.82; −0.71)	−0.94 (−1.31; −0.57)	0.001
Total ruminant trans-fatty acids	1.28 (0.26)	−1.02 (−1.58; −0.47)	−1.35 (−1.90; −0.80)	−0.92 (−1.30; −0.54)	0.001
Industrial trans-fatty acids					
18:1 n-9 elaidic acid	0.14 (0.06)	−0.35 (−0.91; 0.21)	−0.48 (−1.04; 0.08)	−0.29 (−0.64; 0.07)	0.176
n-6 PUFAs					
18:2 linoleic acid	19.65 (3.68)	−0.93 (−1.47; −0.38)	−1.66 (−2.22; −1.11)	−1.11 (−1.46; −0.76)	0.001
18:3 γ-linolenic acid	0.06 (0.04)	−0.32 (−0.88; 0.24)	−0.54 (−1.10; 0.03)	−0.37 (−0.69; −0.06)	0.033
20:2 eicosadienoic acid	0.15 (0.05)	0.09 (−0.46; 0.65)	0.20 (−0.36; 0.75)	0.17 (−0.18; 0.53)	0.410
20:3 dihomo-γ-linolenic acid (DGLA)	2.15 (0.66)	0.70 (0.16; 1.24)	2.35 (1.80; 2.90)	1.86 (1.51; 2.22)	0.001
20:4 arachidonic acid (AA)	8.82 (1.91)	0.58 (0.03; 1.13)	0.95 (0.40; 1.51)	0.67 (0.28; 1.05)	0.002
Total n-6 PUFAs	30.82 (4.20)	−0.32 (−0.88; 0.24)	−0.63 (−1.19; −0.07)	−0.43 (−0.81; −0.04)	0.048
n-3 PUFAs					
18:3 α-linolenic acid	0.04 (0.03)	0.49 (−0.06; 1.04)	1.22 (0.66; 1.77)	0.93 (0.63; 1.22)	0.001
20:5 eicosapentaenoic acid (EPA)	0.64 (0.55)	0.56 (0.00; 1.12)	0.81 (0.25; 1.37)	0.16 (−0.09; 0.41)	0.275
22:6 docosahexaenoic acid (DHA)	2.90 (0.88)	0.06 (−0.51; 0.62)	−0.21 (−0.77; 0.36)	−0.07 (−0.45; 0.30)	0.719
Total n-3 PUFAs	3.57 (1.27)	0.49 (−0.07; 1.05)	0.19 (−0.38; 0.75)	0.08 (−0.27; 0.42)	0.710
Ratio n-6/n-3 PUFAs	9.12 (1.51)	0.22 (−0.34; 0.77)	−0.34 (−0.91; 0.22)	−0.09 (−0.22; 0.04)	0.221

^a^ Mean difference in body mass index comparing Tertile 2 and Tertile 3 with Tertile 1 (reference). ^b^ Mean difference in body mass index comparing women in the 80th percentile with women in the 20th percentile of each fatty acid distribution. ^c^ Adjusted for age, education, smoking, alcohol consumption, physical activity, hypercholesterolemia and energy intake. ^d^ *p* value following Benjamini and Hochberg procedure.

**Table 4:** Association between serum phospholipid fatty acids and anthropometric characteristics.

	Weight Gain Since Age 18			Body Fat Percentage			Visceral Fat Index			Waist Circumference		
	β ^a,b^	SD	q-Val ^c^	β ^a,b^	SD	q-Val ^c^	β ^a,b^	SD	q-Val ^c^	β ^a,b^	SD	q-Val ^c^
SFAs												
14:0 myristic acid	−0.16	0.250	0.918	0.00	0.140	0.998	0.00	0.030	0.935	−0.06	0.270	0.906
15:0 pentadecanoic acid	−0.24	0.250	0.818	−0.12	0.140	0.503	−0.03	0.030	0.388	−0.06	0.260	0.906
16:0 palmitic acid	−0.02	0.260	0.954	0.22	0.150	0.282	0.05	0.030	0.138	0.32	0.220	0.420
17:0 margaric acid	0.06	0.030	0.504	0.01	0.020	0.503	0.00	0.000	0.459	−0.01	0.030	0.890
18:0 stearic acid	−0.10	0.380	0.954	0.19	0.200	0.503	0.06	0.040	0.161	−1.20	0.310	0.003
20:0 arachidic acid	−0.18	0.360	0.918	−0.16	0.190	0.503	−0.01	0.030	0.874	−1.24	0.300	0.003
Total saturates (SFAs)	−0.04	0.360	0.954	0.29	0.190	0.282	0.08	0.030	0.100	−0.86	0.290	0.019
cis-MUFAs												
16:1 n-7 palmitoleic acid	−0.01	0.240	0.954	0.11	0.140	0.503	0.04	0.020	0.182	0.14	0.210	0.790
17:1 heptadecenoic acid	0.06	0.030	0.406	0.01	0.020	0.503	0.00	0.000	0.417	−0.01	0.030	0.906
18:1 n-9 oleic acid	−0.07	0.250	0.954	−0.30	0.140	0.190	−0.06	0.030	0.100	−0.25	0.210	0.506
20:1n-9 gondoic acid	−0.26	0.270	0.818	−0.10	0.150	0.503	−0.04	0.030	0.268	−0.20	0.220	0.668
Total cis-MUFAs	−0.04	0.250	0.954	−0.28	0.140	0.233	−0.05	0.030	0.137	−0.24	0.210	0.515
trans-fatty acids												
Ruminant trans-fatty acids												
16:1 n-7 palmitelaidic acid	−0.46	0.300	0.508	−0.26	0.170	0.282	−0.05	0.030	0.161	−0.13	0.250	0.803
18:1 n-7 vaccenic acid	−0.06	0.310	0.954	−0.21	0.170	0.354	−0.06	0.030	0.133	0.18	0.260	0.790
Total ruminant trans-fatty acids	−0.16	0.320	0.918	−0.26	0.180	0.282	−0.07	0.030	0.100	0.14	0.270	0.803
Industrial trans-fatty acids												
18:1 n-9 elaidic acid	−0.10	0.290	0.954	−0.12	0.160	0.503	−0.03	0.030	0.388	−0.45	0.250	0.241
n-6 PUFAs												
18:2 linoleic acid	−0.26	0.300	0.818	−0.59	0.160	0.007	−0.10	0.030	0.028	0.35	0.250	0.420
18:3 γ-linolenic acid	−0.74	0.260	0.140	−0.42	0.140	0.037	−0.07	0.030	0.065	−0.30	0.220	0.420
20:2 eicosadienoic acid	−0.24	0.290	0.818	0.14	0.160	0.503	−0.02	0.030	0.623	0.24	0.250	0.668
20:3 dihomo-γ-linolenic acid (DGLA)	0.66	0.330	0.401	0.68	0.180	0.007	0.09	0.030	0.065	1.33	0.260	0.003
20:4 arachidonic acid (AA)	0.73	0.320	0.308	0.48	0.180	0.042	0.04	0.030	0.305	1.10	0.260	0.003
Total n-6 PUFAs	0.19	0.320	0.918	−0.22	0.170	0.354	−0.06	0.030	0.138	1.02	0.260	0.003
n-3 PUFAs												
18:3 α-linolenic acid	0.40	0.250	0.508	0.23	0.140	0.282	0.05	0.030	0.137	0.40	0.210	0.232
20:5 eicosapentaenoic acid (EPA)	−0.26	0.210	0.714	0.16	0.110	0.282	0.04	0.020	0.161	−0.02	0.170	0.942
22:6 docosahexaenoic acid (DHA)	−0.27	0.310	0.818	0.27	0.170	0.282	0.00	0.030	0.935	0.15	0.260	0.803
Total n-3 PUFAs	−0.32	0.290	0.803	0.28	0.160	0.282	0.03	0.030	0.459	0.10	0.240	0.868
Ratio n-6/n-3 PUFAs	0.04	0.100	0.954	−0.07	0.060	0.372	−0.01	0.010	0.415	0.07	0.090	0.686

^a^ Mean difference of each anthropometric variable comparing women in the 80th percentile with women in the 20th percentile of each fatty acid distribution. ^b^ Adjusted for age, education, body mass index, smoking, alcohol consumption, physical activity, hypercholesterolemia and energy intake. ^c^ *p* value following Benjamini and Hochberg procedure.

**Table 5:** Association between desaturation indices and anthropometric characteristics.

	Body Mass Index			Weight Gain Since Age 18			Body Fat Percentage			Visceral Fat Index			Waist Circumference		
	β ^a,b^	SD	q-Val ^d^	β ^a,c^	SD	q-Val ^d^	β ^a,c^	SD	q-Val ^d^	β ^a,c^	SD	q-Val ^d^	β ^a,c^	SD	q-Val ^d^
**Desaturation indices**															
SCD-16: 16:1n-7c/16:0	0.51	0.16	0.001	0.00	0.250	0.993	0.08	0.140	0.575	0.03	0.030	0.237	0.07	0.210	0.738
SCD-18: 18:1n-9c/18:0	−0.95	0.17	0.001	0.04	0.290	0.993	−0.17	0.160	0.425	−0.04	0.030	0.237	0.56	0.240	0.038
FADS1: 20:4n-6/20:3n-6	−1.02	0.16	0.001	−0.08	0.270	0.993	−0.15	0.150	0.425	−0.02	0.030	0.441	−0.39	0.230	0.113
FADS2: 20:3n-6/18:2	2.24	0.18	0.001	0.75	0.330	0.092	0.88	0.180	0.002	0.13	0.030	0.002	1.04	0.270	0.002

^a^ Mean difference of each anthropometric variable when comparing women in the 80th percentile with women in the 20th percentile of each desaturation index. ^b^ Adjusted for age, education, smoking, alcohol consumption, physical activity, hypercholesterolemia and energy intake. ^c^ Adjusted for age, education, body mass index, smoking, alcohol consumption, physical activity, hypercholesterolemia and energy intake. ^d^ *p* value following Benjamini and Hochberg procedure.
